# Development of Hydroxyapatite/Polycaprolactone Composite Biomaterials for Laser Powder Bed Fusion: Evaluation of Powder Characteristics, Mechanical Properties and Biocompatibility

**DOI:** 10.3390/polym16060731

**Published:** 2024-03-07

**Authors:** Hongbo Yao, Wei Zhu, Xiaolong Zhu, Xiner Yi, Jinchao Yao, Xun Yuan, Feng Chen, Xiaoxiao Han

**Affiliations:** 1 National Engineering Research Centre for High Efficiency Grinding, College of Mechanical and Vehicle Engineering, Hunan University, Changsha 410082, China; yaohongbo@hnu.edu.cn (H.Y.); xiaolz@hnu.edu.cn (X.Z.); ironman@hnu.edu.cn (J.Y.); xunyuan@hnu.edu.cn (X.Y.); fchen1101@outlook.com (F.C.); 2 Xiangya School of Pharmacy, Central South University, Changsha 410083, China; 8202210205@csu.edu.cn

**Keywords:** additive manufacturing, selective laser sintering, polycaprolactone, hydroxyapatite, bone tissue engineering

## Abstract

Hydroxyapatite/polycaprolactone (HA/PCL) composites have been extensively explored in laser powder bed fusion (L-PBF) for bone tissue engineering. However, conventional mechanical mixing methods for preparing composite powders often yield inhomogeneous compositions and suboptimal flowability. In this study, HA/PCL powders were prepared and optimized for L-PBF using the modified emulsion solvent evaporation method. The morphology, flowability and thermal and rheological properties of the powders were systematically investigated, along with the mechanical and biological properties of the fabricated specimens. The HA/PCL powders exhibited spherical morphologies with a homogeneous distribution of HA within the particles. The addition of small amounts of HA (5 wt% and 10 wt%) enhanced the processability and increased the maximum values of the elastic modulus and yield strength of the specimens from 129.8 MPa to 166.2 MPa and 20.2 MPa to 25.1 MPa, respectively, while also improving their biocompatibility. However, excessive addition resulted in compromised sinterability, thereby affecting both mechanical and biological properties.

## 1. Introduction

Large bone defects resulting from accidents, trauma, osteoarthritis, tumors, and severe comminuted fractures often require intervention as they cannot heal spontaneously. The utilization of autograft and allograft transplantation, as conventional therapeutic modalities for this condition, is significantly constrained by the scarcity of donors, complications, and immune rejection reactions [1,2]. Bone tissue engineering (BTE) holds immense potential in the treatment of large bone defects. It is anticipated that through the implantation of artificial bone scaffolds seeded with cells and growth factors at the site of defects, flawless restoration of the bone tissue defect can be achieved [3,4,5,6]. The development of novel biomaterials and manufacturing methods for biomimetic artificial bone scaffolds, which play a pivotal role in the restoration of bone defects, has attracted tremendous research attention.

Polymeric materials are commonly used for scaffold fabrication in BTE due to their excellent formability, biocompatibility, and tunable mechanical strength [7,8]. Several biocompatible polymeric materials, such as polycaprolactone (PCL) [9,10,11,12], polylactic acid (PLA) [13,14,15], polyglycolic acid (PGA) [13,16,17], polyurethane (PU) [18], and polyether ketone (PEEK) [19], have been extensively studied and employed for the production of scaffolds. PCL, a synthetic and biodegradable polymer, has gained approval from both the U.S. Food and Drug Administration and the European Medicines Agency for its applications in healthcare. With its excellent solubility, low melting point, flexibility, minimal degradation byproducts, and extended degradation time of 1~2 years [20], PCL has attracted significant attention in the fields of BTE, long-term surgical implants, and slow-release drug delivery systems [21,22,23]. However, the hydrophobicity and low bioactivity of PCL impede cell adhesion on the scaffold and exert an impact on cell proliferation and differentiation, thereby decelerating the process of bone defect repair [24].

To attain enhanced bioactivity and hydrophilicity of polymer materials, composite biomaterials have been developed by incorporating an inorganic phase, such as bioceramics that mimic the composition of natural bone [2]. This allows for harnessing the synergistic advantages offered by multiple materials [25]. Calcium phosphate ceramics such as β-tricalcium phosphate (β-TCP), bioglasses, and hydroxyapatite (HA) are widely used bioceramics. Among these, HA is the primary inorganic component found in human bones and possesses excellent biocompatibility and osteoinductive capabilities. Consequently, composites composed of PCL and HA have emerged as ideal materials for scaffolds used in BTE [26,27,28,29].

Generally, the irregularity of geometric shape in bone defects and the unique nature of each patient’s defect necessitate individualized solutions for implant design and manufacturing. Additive manufacturing (AM) technology, as a layer-wise manufacturing method, is superior to traditional manufacturing methods (such as injection molding and CNC machining) in creating personalized implants [30,31]. Selective laser sintering (SLS), involving laser powder bed fusion (L-PBF) of the AM process, utilizes a laser as the heat source to sinter powdered materials selectively. It is widely regarded as one of the most established and extensively utilized AM processes for polymer processing. Compared to other AM technologies, SLS eliminates the need for additional support structures during the printing process. This capability enables SLS to fabricate structures with particularly complex shapes [32], which confers significant advantages in bone repair and regeneration.

SLS technology has been employed in previous studies to manufacture PCL and its composites. Williams et al. pioneered the utilization of SLS technology in fabricating scaffolds using PCL materials that closely matched the mechanical properties of trabecular bone. Additionally, CT scanning revealed promising outcomes for using SLS in manufacturing mandibular models and bone scaffolds [33]. Huang et al. utilized SLS to manufacture scaffolds using a mixed powder of PCL and sodium chloride as a pore-forming agent. Subsequent removal of the sodium chloride resulted in a liver tissue scaffold characterized by intricate 3D flow channels and a high porosity of 89%. The biological results demonstrated that these 3D flow channels significantly promote cell growth and functional expression [34]. Doyle et al. fabricated composite scaffolds comprising PCL and β-TCP through SLS, which exhibited a decrease in both elastic modulus and strength, as well as the alteration of degradation properties upon the incorporation of β-TCP [35]. Hollister et al. conducted a comprehensive study on the utilization of SLS for manufacturing PCL tracheal scaffolds, which were employed in a clinical trial for the treatment of pediatric patients with tracheobronchial cartilage collapse [36,37]. The results indicated that the scaffold’s opening stiffness of 4 mm/N provides sufficient support for tracheal growth [38]. Liu et al. prepared HA/PCL scaffolds loaded with vascular endothelial growth factor (VEGF), which exhibited excellent biocompatibility and promoted osteogenic differentiation of stem cells, leading to a significant enhancement in vascularization and bone generation in vivo [39].

The conventional approach for preparing composite biomaterial powders involves mechanically crushing PCL powder followed by incorporating HA particles directly into the powder [38,40,41,42]. However, this method often results in an uneven distribution of HA, leading to poor flowability, reduced precision, and compromised and inconsistent mechanical properties of the scaffolds, which are critical factors for the application of biomedical devices. Du et al. introduced the emulsification solvent evaporation method to synthesize HA/PCL composite microspheres, which were subsequently used in the fabrication of scaffolds via SLS [43,44]. Similar approaches have also been applied in other relevant investigations. Liu et al. used this method to fabricate the scaffold for vascularized bone regeneration [39,43,44,45]. In addition, Gu et al. used this method to fabricate scaffolds for osteochondral repair [45]. This method can improve the uniformity of HA and the precision of scaffold fabrication. However, current research primarily focuses on the scaffold structure and the influence of materials on the biological functionality of the scaffold. The preparation and optimization of HA/PCL composite microsphere powders and their effect on printability (flowability, thermal behavior, and rheology properties), mechanical performance, and biocompatibility of the SLS-processed scaffolds have not been thoroughly explored.

In this study, the HA/PCL composite biomaterial powders amenable to SLS were prepared using the modified emulsion solvent evaporation method. The morphology, flowability, thermal behavior, and rheology properties of the prepared powders were characterized. Subsequently, specimens were manufactured through SLS with a wide range of printing parameters, and compression testing and porosity measurements were conducted to determine variations in mechanical performance and optimize the processing parameters. Finally, biocompatibility experiments were performed on the scaffolds to validate their feasibility for BTE applications.

## 2. Materials and Methods

### 2.1. Preparation of Powder by Solvent Evaporation

**Figure 1:**
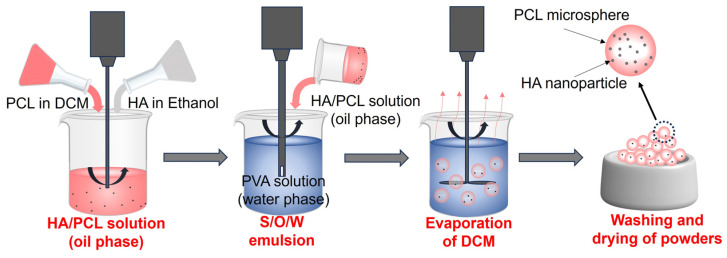
Schematic for preparing HA/PCL composite powders using the modified emulsion solvent evaporation method.

PCL (Mn = 50,000) was commercially purchased in a powdered form with an average particle size of 250 µm. HA nanoparticles (SKU: H106378) with a size of 100 nm were purchased from Aladdin Shanghai. Pure PCL and HA/PCL powders were prepared using a modified solid-in-oil-in-water (S/O/W) emulsion solvent evaporation method. First, 50 g of PCL was dissolved in 300 mL of dichloromethane (DCM). Subsequently, various concentrations of HA (2.5 g, 5 g, and 10 g) were added to 30 mL of ethanol and agitated for 30 s using an emulsifier to ensure thorough dispersion. Then, the ethanol solution was introduced into the DCM solution and stirred to generate a suspension, which was then added to 1500 mL of polyvinyl alcohol (PVA) solution (1 *w*/*v*%) and vigorously stirred for 5 min at 9000 rpm using an emulsifier to form an emulsion. After that, the emulsion was stirred continuously for 6 h at 25 °C to ensure the evaporation of DCM. Finally, the resulting powder was collected, subjected to 3 rounds of washing with pure water, and subsequently dried for further processing. The process of the preparation of powder is shown in Figure 1. The HA/PCL composite powders with varying ratios of HA were prepared, namely PCL, 5HA/PCL, 10HA/PCL, and 20HA/PCL powders representing pure PCL and powders incorporating a 5%, 10%, and 20% weight ratio of HA, respectively.

### 2.2. Powder Characterization

The particle size and distribution of the powders were tested using a laser particle analyzer (LS-POP9, OMEC). The span of the particle size distribution was calculated using (D_90_ − D_10_)/D_50_. D_n_ is the particle size that a cumulative percentage of the particle size distribution in a powder sample corresponds to when it reaches n%. [46] The surface morphology of the powders was observed using scanning electron microscopy (SEM) with an MIA3 from TSCAN at an energy of 5 KeV. The powder flowability was evaluated according to the Chinese standard GB/T 31057.3-2018 using a BT-1000 powder characterization tester from Bettersize Instruments (Dandong, China) [47]. For measuring the tap density, the frequency of vibration was set as 250 vibrations per minute and the amplitude was 3 mm. The Hausner ratio (HR) of the powders was then obtained by dividing the tap density values by the bulk density values. Four parameters related to the followability (i.e., angle of repose, angle of spatula, compressibility, and uniformity coefficient) can also be obtained through the BT-1000 instrument. Each measurement can be converted into a corresponding index number (scored out of 25) using the Carr Flowability Chart [48], and the sum of these four index numbers is defined as the flowability index. By referring to the flowability index obtained from the chart, the severity of either bridging or compaction of a material can be determined (Table S1).

Differential scanning calorimetry (DSC) tests were conducted using a DSC3 from Mettler Toledo. The powder was subjected to a heating process in a nitrogen atmosphere from 20 °C to 100 °C at a controlled rate of 10 °C/min; subsequently, it was cooled back to 20 °C at the same rate. The starting melting temperature *T*_im_, peak melting temperature *T*_m_, starting crystallization temperature *T*_ic_, and peak crystallization temperature *T*_c_ were obtained from these tests.

Thermogravimetric analysis (TGA) was carried out using a TGA209F1 instrument from NETZSCH. The powders were heated from 20 °C to 500 °C at a rate of 10 °C/min in a nitrogen atmosphere. The temperature at which the powder produced a 1% mass loss was determined as the thermal degradation onset temperature *T*_d,oneset_. The sample quantities utilized for DSC and TGA analyses ranged from 5 mg to 10 mg, with three repetitions conducted for each sample.

The rheological behavior and melt viscosity of various powders were evaluated using the Kinexus pro+ instrument from Malvern Panalytical. The powder was loaded onto a 40 mm-diameter parallel plate with a 1 mm gap between plates and subjected to oscillatory shear testing at 85 °C with an angular velocity range of 0.1–100 rad/s. All tests were repeated three times for each material.

### 2.3. Specimen Preparation and Characterization

The specimens were fabricated using a SnowWhite2 SLS 3D printer from Sharebot, Nibionno, Italy. The geometries of the cylindrical specimens for compression tests are Φ10 mm × 5 mm according to the standard ISO 604:2002 [49]. The cylinder specimens used for cell culturing have a geometric dimension of Φ5 mm × 2 mm. The optimization for process parameters was conducted for each material by varying the laser power with an interval of 0.7 W while keeping other parameters constant. The specific processing parameters are shown in Table 1. The laser energy was calculated using *p*/*sht* [50].

Compression tests were conducted at room temperature using an AGS-X electronic universal testing machine from Shimadzu. The loading rate was set to 5 mm/s for all specimens. The second derivative of the stress–strain curve is used for analyzing the yield strength of the specimen, which corresponds to the zero point (inflection point) of the second-order derivative of the curve. The porosity was determined using the Archimedes’ method with a precision balance and density test kit. For all the mechanical tests and porosity measurements, at least 3 specimens were tested, and the resulting values were averaged.

### 2.4. In Vitro Cell Culture to Evaluate the Biocompatibility of Scaffolds

The biocompatibility of PCL-based scaffolds fabricated using SLS was assessed through in vitro culture with human osteosarcoma cells (MG-63). The MG-63 cells were cultured in 1640 medium containing 10% *v*/*v* fetal bovine serum (FBS, Gibco) and 1% (*v*/*v*) penicillin–streptomycin–amphotericin B (PSA, Gibco). All the scaffolds were sterilized with ethanol for 30 min and irradiated with UV light overnight. Subsequently, the sterilized scaffolds were infiltrated with a culture medium overnight before cell seeding. The cell adhesion performance on scaffolds was evaluated by seeding 50,000 cells per well onto the surface of the scaffold in 96-well plates. The cytoskeleton and nucleus were stained with phalloidin-iFluor 488 (Biolite, China) and 2-(4-Amidinophenyl)-6-indolecarbamidine dihydrochloride (DAPI, 1:1000, Beyotime, China), respectively, the following day. The cell morphology on scaffolds was observed with fluorescence microscopy (Olympus, Japan). In addition, the cells on the scaffold were fixed and dehydrated with a gradient concentration of alcohol (30%, 50%, 70%, and 100%) before being placed under the SEM for observation. The cell proliferation was assessed by culturing the cells at a density of 10,000 per well on the scaffold surface in 96-well plates, and the cell activity was evaluated using Cell Counting Kit-8 (CCK-8, Meilunbio, Dalian, China) on day 1, day 3, day 5, and day 7.

## 3. Results and Discussion

### 3.1. Powder Morphology and Flowability

**Figure 2:**
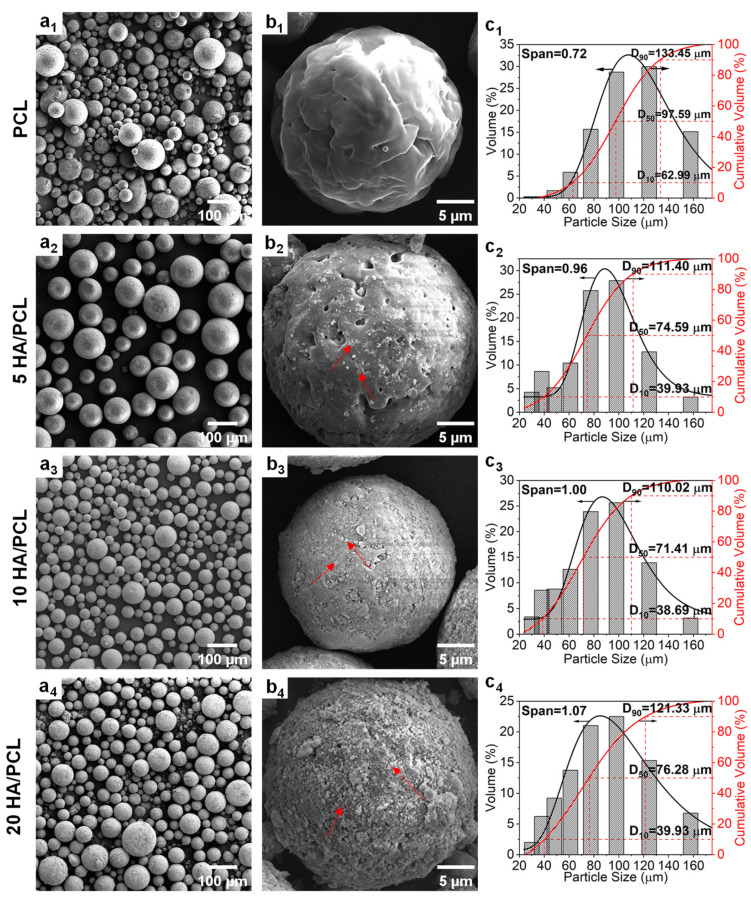
(**a_1_**–**a_4_**,**b_1_**–**b_4_**) SEM images and (**c_1_**–**c_4_**) particle size distribution curves of the PCL and HA/PCL composite powders ((**b_1_**–**b_4_**) are the high-magnification images; red arrows point to HA particles).

The particle morphology and size of powders exert a significant impact on their flowability, thereby influencing the surface roughness and strength of the produced parts. The ideal powders for SLS should have a narrow particle size distribution and preferably exhibit a spherical morphology [51]. The SEM images confirmed that the prepared pure PCL powder as well as the 5HA/PCL, 10HA/PCL, and 20HA/PCL composite powders demonstrated a relatively optimal spherical morphology (Figure 2a_1_–a_4_). PCL exhibited a round shape but a less smooth surface due to the presence of numerous wrinkles and micropores. This phenomenon could be attributed to the reliance of emulsion on interfacial tension for maintaining a spherical shape. As the solvent evaporated, PCL initiated crystallization at the spherical interface, resulting in a process of crystalline bending that generates elastic potential energy. Consequently, defects and pores were formed within the crystals to release this stored energy [52,53]. White HA particles (marked with red arrows) were uniformly distributed on the surface (Figure 2b_2_–b_4_); compared to the mechanical mixing method, the powder prepared in this study allowed a more homogeneous HA distribution while avoiding the sedimentation of nanoparticles. The pores and defects on the microsphere surface decreased with the increase in HA. In contrast to the surface morphology of PCL microspheres, HA particles filled the pores and defects that covered the microsphere formation process. However, as the amount of HA increased, HA produced aggregation on the microsphere surface, making the microsphere surface rougher and thus affecting the flowability of the powder.

The particle size distributions of the PCL powders are presented in Figure 2c_1_–c_4_. Overall, all the powders exhibited a particle size within the range of 40~130 μm, with the pure PCL showing larger particles in the range of 60~133 μm. Additionally, the median diameter (D_50_) of the HA/PCL composite powders was approximately 70 μm, except for pure PCL, which had a D_50_ value of 98 μm. Upon the addition of HA, there was a decrease in powder D_50_ and an increase in span value due to the heterogeneous nucleation induced by HA during the solvent evaporation process. The presence of HA particles accelerated the droplet crystallization rate while simultaneously impeding crystal growth, resulting in the formation of more fine particles compared to pure PCL. As the amount of HA increased, the crystallization induced by the aggregated HA led to larger microspheres and, consequently, a wider size distribution. This phenomenon explained the observed increase in span value with increasing HA content. Nevertheless, the method used in this study enables convenient and flexible adjustment of the average particle size distribution through manipulation of processing parameters such as the PCL concentration and emulsification speed (Figure S1).

The angle of repose serves as a direct indicator of powder flowability, whereby a smaller angle indicates superior powder fluidity. According to Carr’s criteria for fluidity classification, an angle of repose below 30° signifies good flowability [54]. The addition of HA resulted in a decrease in the angle of repose from 35.2° for PCL powder to below 30°, indicating an improvement in flowability (Table 2).

The Hausner ratio (HR) is a representative flow characteristic of powders, which is calculated as the ratio between tap density and bulk density. Powders with an HR < 1.25 are considered to have good flowability and are suitable for SLS [56]. As shown in Table 2, the HR value of PCL was 1.158, which is comparable to the widely used commercial polyamide 12 (PA12) powder in PBF [55]. The incorporation of HA particles resulted in a decrease in the HR values for 5HA/PCL and 10HA/PCL to 1.045 and 1.058, respectively, indicating their excellent flowability. With a further increase in the amount of HA, the HR of 20HA/PCL increased to 1.203. The results of the flowability index, which provided a more comprehensive and well-rounded assessment of the powder flowability, further substantiated the observation. With an appropriate amount of HA (5% and 10%), the nanoparticles on the PCL surface could function as a lubricant, reducing the interparticle forces [57]. However, excessive HA (20%) led to particle aggregation and hindered the powder flow. These findings aligned with the SEM analysis of surface morphology.

### 3.2. Thermal Properties

#### 3.2.1. Melting and Crystallization

**Figure 3:**
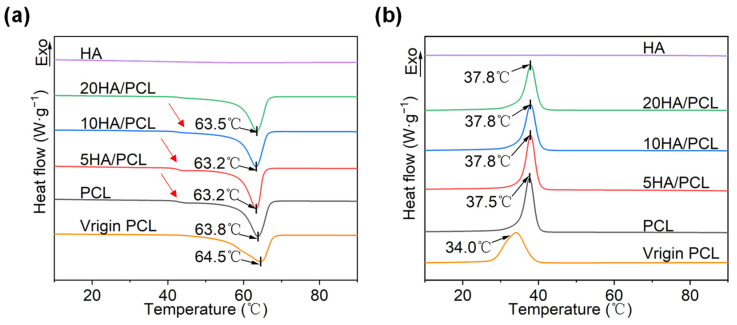
DSC curves of the virgin PCL, PCL, and HA/PCL composite powders: (**a**) heating and (**b**) cooling process at a rate of 10 °C/min.

The DSC curves for the heating and cooling process of each material are shown in Figure 3, where virgin PCL is the raw material directly obtained from the supplier for powder preparation. HA did not show any endothermic or exothermic behavior within this temperature range, and all the curves reflected the melting and crystallization characteristics of PCL. In Figure 3a, the melting temperature *T*_m_ of the powders remained essentially unchanged, while a significant disparity was observed in the melt onset temperature *T*_im_ between the virgin PCL and the prepared powders. The discrepancy could be attributed to the commercial powders often exhibiting heterogeneity and containing additives, whereas powders made using solvent evaporation allowed for further purification of the PCL material. In addition, the width of the melting peak suggested that crystallization from the solvent provided a prolonged time for the PCL chains to rearrange and form a concentrated thickness distribution of thicker crystal lamellae, thus increasing the melt onset temperature [58,59]. This also contributed to the observation that the prepared powders exhibited narrower and more distinct peaks compared to those of virgin PCL (Figure 3a). In addition, as shown in Figure 3a, the prepared PCL powder had a low-temperature shoulder (indicated by the red arrows) at around 40 °C, which gradually decreased with the addition of HA. This phenomenon could be attributed to the evaporation of the solvent during the formation of microspheres. As the solvent evaporated, the polymer shell underwent continuous contraction, leading to its fragmentation into small pieces, acting as nuclei for the secondary crystallization of PCL [60]. However, the addition of HA particles resulted in the inhibition of secondary crystallization [61], as evidenced by the observed decrease in enthalpy for the low-temperature shoulder in heating curves for HA/PCL composite powders (Figure 3a). The cooling cycles of the PCL materials are depicted in Figure 3b, wherein a slight increase in the crystallization onset temperature T_ic_ and a reduction in the degree of crystallinity x_c_ of PCL were observed (Table 3). These findings suggested that the addition of HA showed a noticeable nucleation effect on the molten PCL [62]. This could be attributed to HA hindrance on the mobility of PCL molecular chains [63]. However, there was little difference in crystallinity, indicating that substantial alterations in laser energy are unnecessary during the SLS process.

The sintering window of the material, which is defined as the difference between the melting onset temperature *T*_im_ and the crystallization onset temperature *T*_ic_, plays an essential role in determining the sinterability. Based on Figure 3b and Table 3, the sintering windows of PCL, 5HA/PCL, 10HA/PCL, and 20HA/PCL were determined to be 19.1 °C, 18.6 °C, 18.1 °C, and 18.2 °C, respectively, all wider than that of the virgin PCL powder (15.4 °C). The expanded sintering window could be attributed to the large enhancement in *T*_im_ and negligible alteration in the *T*_ic_ of the prepared powders, indicating an improvement in sinterability. Furthermore, the sharper melting peaks of the prepared powders (shown in Figure 3a), in comparison to virgin PCL material, contributed to improved powder flowability at high temperatures. Simultaneously, this would help to minimize excessive sintering of the particles beyond the scanning boundaries, thereby improving the contour resolution of the fabricated parts [64].

#### 3.2.2. Thermal Stability

**Figure 4:**
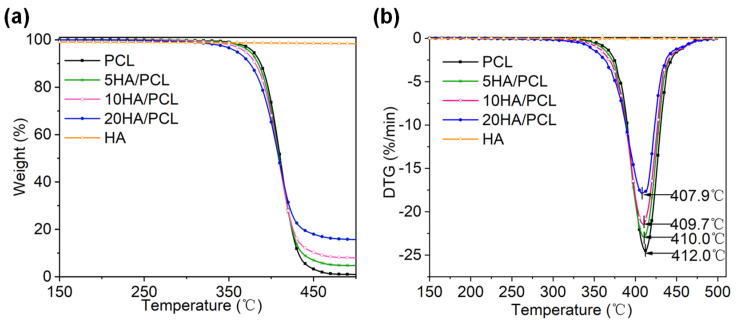
Thermal stability of the PCL and HA/PCL composite powders under air atmosphere: (**a**) TG and (**b**) DTG curves.

The thermal stability of the biomaterials for SLS processing is crucial, given the prolonged duration of the heating process. The thermogravimetric (TG) (Figure 4a) and derivative thermogravimetry (DTG) (Figure 4b) data indicated that HA did not degrade below 500 °C. Among all the prepared powders, an increase in HA content led to a decrease in the onset degradation temperature *T*_d,onset_ and maximum degradation temperature *T*_d,max_, as shown in Table 4. The reduction in thermal stability could be attributed to the aggregation of HA within the microspheres, which lowered the activation energy barrier. Additionally, previous studies have also indicated that the addition of calcium phosphate tends to induce polymer chain breakage [65,66]. Conversely, the presence of HA on the microsphere surface hindered the degradation and spillover of volatile decomposition substances, resulting in a decrease in *r*_d,max_ as the HA content increased. It is worth noting that all materials exhibited significantly higher degradation temperatures than their melting temperatures, indicating sufficient thermal stability of the composite materials during processing. The residual mass at 500 °C of PCL, 5HA/PCL, 10HA/PCL, and 20HA/PCL was determined to be 1.01%, 3.9%, 7.5%, and 17.2%, respectively. The measured content of HA in the PCL composite powder was lower than the theoretical values, indicating a slight loss of HA during the powder preparation process. The loss of HA probably occurred in the process of transferring HA dispersion into the aqueous solution and subsequent solvent evaporation, where HA entered the aqueous phase from the emulsion interfaces due to its hydrophilic nature.

### 3.3. Rheological Properties

**Figure 5:**
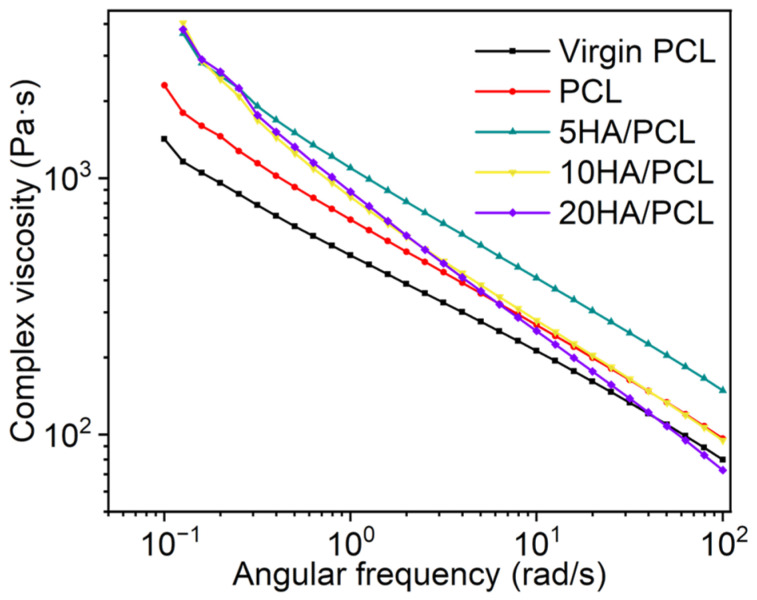
Complex viscosity curves of virgin PCL, PCL, and HA/PCL composite powders.

The complex viscosity characteristics of various materials at 85 °C are shown in Figure 5. All the composite powders exhibited non-Newtonian and shear-thinning behavior. The viscosity of the HA/PCL composite powders was higher than that of PCL, primarily attributed to the presence of nanoparticles that impeded the movement of the molecular chains and thereby increased the melt viscosity. Additionally, the abundant -OH groups in HA could form hydrogen bonds with PCL [67]. The difference in viscosity between 5HA/PCL, 10HA/PCL, and 20HA/PCL at a low shear rate was not significant. However, an increase in HA content resulted in a rapid decrease in viscosity at high rates. The composite powders with a higher HA content exhibited elevated shear stress as the shear rate increased, leading to more rapid decreases in viscosity [68].

According to the Frenkel–Eshelby viscosity model, surface tension and zero-shear viscosity (η_0_) are the primary factors affecting the coalescence behavior of powder particles in the SLS process [69]. A lower η_0_ leads to a higher rate of consolidation. To obtain the material η_0_, the Carreau–Yasuda model was used to analyze the complex viscosity of PCL and HA/PCL composites as a function of frequency, as it not only describes the Newtonian behavior of the fluid at low shear rates but also captures the shear thinning process at high shear rates. The complex viscosity η of polymers is generally described using the Carreau–Yasuda model [70]:

(1) $$\eta =\eta_{\infty }+\left(\eta_{0}-\eta_{\infty }\right){\left[1+{\left(\lambda \omega \right)}^{a}\right]}^{\left(n-1\right)/a}$$

where η_∞_ is the infinite shear viscosity, η_0_ is the zero-shear viscosity, λ is the relaxation time, *n* is the power law index, and *a* is the width of the transition region between Newtonian and power law behavior. The zero-shear viscosities of virgin PCL, PCL, 5HA/PCL, 10HA/PCL, and 20HA/PCL were determined to be 3304.3 Pa·s, 5522.7 Pa·s, 7817.6 Pa·s, 9092.6 Pa·s, and 8177 Pa·s, respectively (the fitting parameters are presented in Table S2). Although these values seem relatively high compared to PA2200, they were comparable to the viscosity of commercial PA12 Orgasol, which has a viscosity of 8000 Pa·s at temperatures above its melting point of 25 °C [71]. With appropriate printing strategies, the prepared powders could be effectively utilized in powder bed manufacturing processes.

### 3.4. Mechanical Properties of the Scaffolds

**Figure 6:**
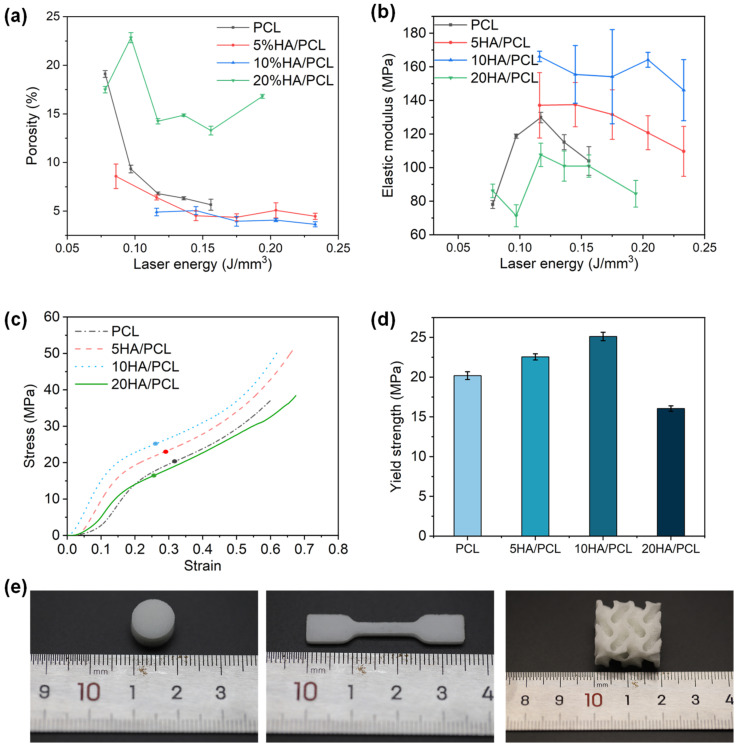
Mechanical properties of the SLS-manufactured scaffolds: (**a**) porosity; (**b**) compressive modulus; (**c**) typical stress–strain curves of the scaffolds at the laser energy of 0.12 J/mm^3^ (the points in the figure are the corresponding yield strengths); (**d**) yield strength at the laser energy of 0.12 J/mm^3^. (**e**) Photos of typical specimens.

The porosity and mechanical properties of the fabricated PCL and HA/PCL composite specimens are shown in Figure 6. Tissue engineering scaffolds should have a specific porosity for the exchange of substances and waste materials in cell culture while also providing sufficient compression strength to support new bone regeneration [72,73]. In this study, at least five different laser energy levels were used for SLS printing of the PCL and HA/PCL composite specimens to explore the properties of the fabricated parts and optimize processing parameters. Additionally, slight adjustments were made to the laser energy range during printing to ensure the successful fabrication of the specimens.

The porosity of all materials decreased with increasing laser energy and then stabilized within a certain range (Figure 6a). This was because higher laser energy promoted powder sintering, reducing interparticle voids and decreasing porosity. However, when the material approached densification, a further increase in laser energy led to over-sintering, preventing further reductions in the porosity. The porosity of the specimens ranged from 3.6% to 22.8% depending on the processing parameters. Compared to PCL, 5HA/PCL and 10HA/PCL showed lower porosity at the same level of laser energy, potentially attributed to the enhanced energy absorption resulting from the presence of HA on the powder surface. However, among all of the materials tested, 20HA/PCL specimens showed the highest porosity due to the significant amount of HA on the microspheres’ surface (Figure 2b_4_), which hindered the coalescence of the adjacent particles and ultimately increased the porosity.

The specimens were subjected to compression tests to evaluate their elastic modulus and yield strength. The elastic modulus of pure PCL and 20HA/PCL specimens initially increased with the rise in laser energy, reaching a maximum of 129.8 MPa and 107.6 MPa, respectively, at a laser density of 0.12 J/mm^3^ due to the improved densification (Figure 6b). Subsequently, the elastic modulus decreased due to polymer degradation at high energy levels. However, for the 5HA/PCL and 10HA/PCL specimens, the modulus exhibited a decreasing trend as the laser energy exceeded 0.12 J/mm^3^, with the respective maximum elastic modulus of 137.5 and 166.2 MPa. This was due to the fact that this relatively low laser energy density was adequate in ensuring a comparatively high density. Further escalation of energy input compromised the elastic modulus. It was also notable that the elastic modulus of the HA/PCL composite generally had a higher elastic modulus than the pure PCL specimens, with the exception being the 20HA/PCL specimen. Furthermore, the 5HA/PCL and 10HA/PCL specimens showed improved yield strength to 22.5 and 25.1 MPa, respectively, compared to the pure PCL specimens (20.2 MPa) fabricated at the same energy density of 0.12 J/mm^3^, while the yield strength of the 20HA/PCL specimen decreased to 16.1 MPa (Figure 6d). This observation aligned with the results of the porosity and elastic modulus. The modulus of the prepared HA/PCL scaffolds indicates their suitability for bone tissue repair in non-load-bearing bones such as trabecular bone, which typically exhibits a modulus ranging from 60 to 440 MPa [74].

**Figure 7:**
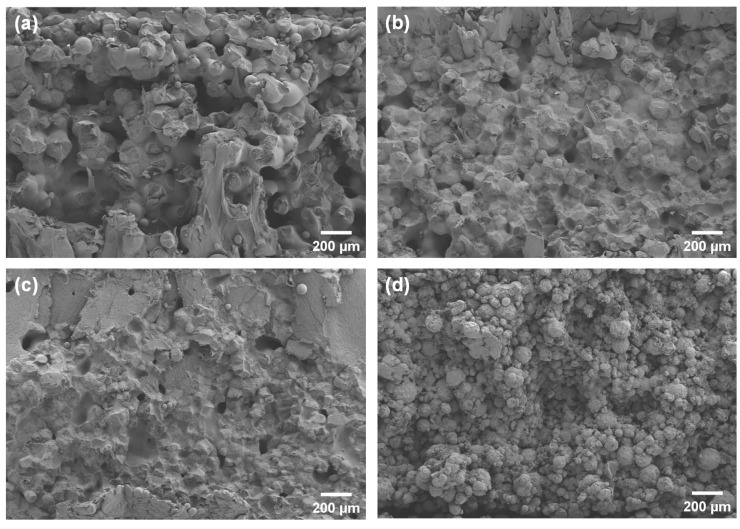
SEM images of cross-sections of the SLS specimens fabricated at 0.12 J/mm^3^: (**a**) PCL; (**b**) 5HA/PCL; (**c**) 10HA/PCL; (**d**) 20HA/PCL.

The SEM images showed cross-sections of the specimens fabricated at the same laser energy of 0.12 J/mm^3^ (Figure 7). The presence of distinct interlayer pores was observed in pure PCL, while the 5HA/PCL specimens exhibited a granular structure. In contrast, the 10 HA/PCL specimens displayed an enhanced degree of sintering, whereas the 20HA/PCL samples showed loosely packed individual microspheres with limited sintering necks. This observation further supported the hypothesis that a judicious amount of HA enhanced the energy absorption capacity of the powder, thereby facilitating sintering, whereas an excessive amount of HA impeded the sintering process and led to high porosity and inferior mechanical properties.

### 3.5. Biocompatibility of the Scaffolds

**Figure 8:**
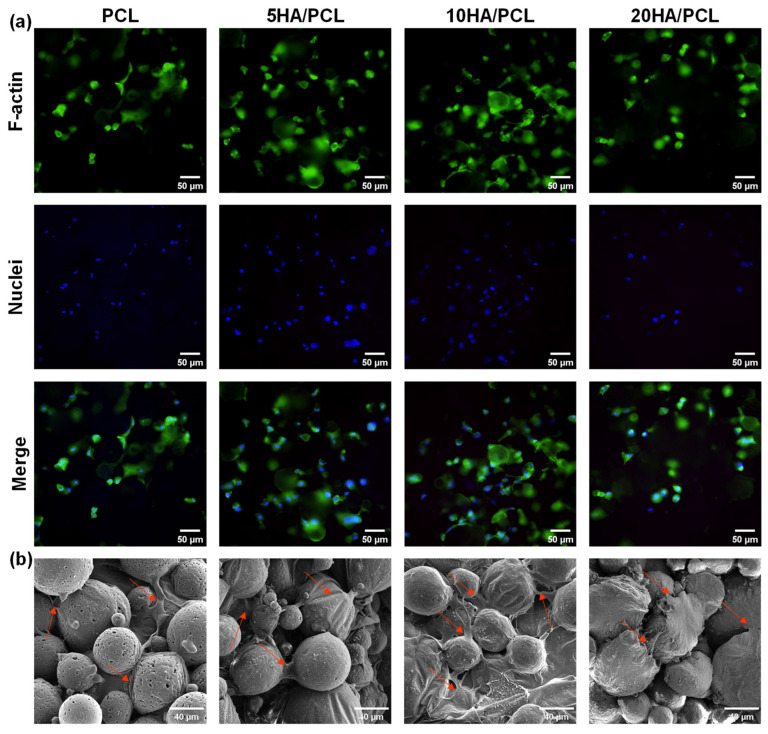
(**a**) Fluorescent staining image of MG-63 on the scaffold, F-actin stained with phalloidin (green), cell nuclei stained with DAPI (blue); (**b**) SEM image of MG-63 cells on the scaffold after 24 h (arrows point to cells).

The biocompatibility of the SLS-fabricated PCL-based scaffolds was evaluated through in vitro cell culture. The adhesion of the MG-63 cells on the scaffolds after 24 h was observed using a fluorescent microscope, images from which are shown in Figure 8a. The cell nuclei were stained blue using DAPI and exhibited a distributed pattern, forming a network distribution surrounding the microspheres on the outer surface of the scaffolds. The F-actin (stained green) also displayed a similar distribution around these spherical areas, with some connections observed between them, indicating the adhesion of the cytoskeleton on the surface of the microspheres within the scaffolds. The SEM analysis further revealed the adhesion of MG-63 cells onto the surface of the microspheres and within the interstitial spaces, with concurrent extension of filopodia to neighboring microspheres (Figure 8b). These results indicated that the inherent rough top surface of the SLS-printed scaffolds provided ample microspheres and pores, which offered a substantial surface area for cell attachment, growth, and migration. Among the various scaffolds tested, the 5HA/PCL and 10HA/PCL scaffolds demonstrated enhanced cell attachment and spreading compared to pure PCL; however, a further increase in HA content resulted in a decrease in both the growth and migration of MG-63 cells.

**Figure 9:**
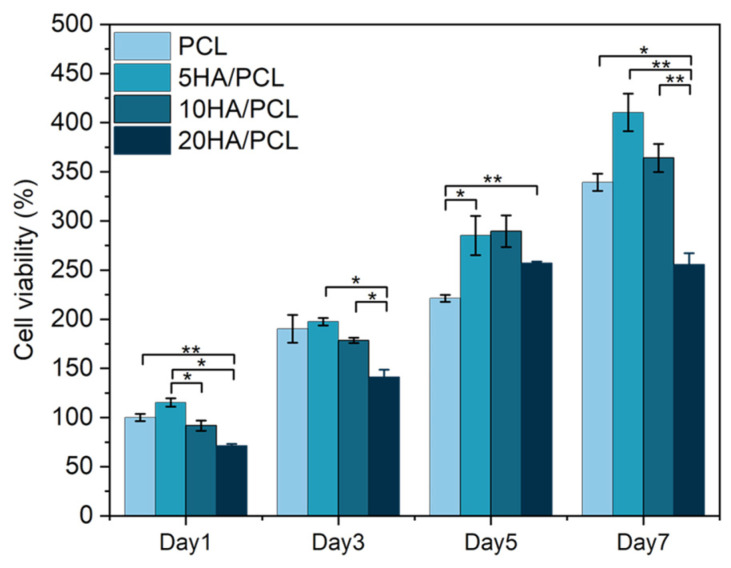
Cell proliferation on the PCL and HA/PCL composite scaffolds on days 1, 3, 5, and 7 (*n* = 5, * indicates *p* < 0.05, ** indicates *p* < 0.01).

The proliferation of MG-63 cells on the SLS-manufactured scaffolds with different HA ratios, as evaluated using the CCK-8 assay on days 1, 3, 5, and 7, is illustrated in Figure 9. The results demonstrated that the cell viability of all the scaffolds increased over time, indicating the excellent biocompatibility of the scaffolds fabricated using PCL and HA/PCL composite powders. Furthermore, in comparison to the PCL scaffold, the 5HA/PCL scaffold exhibited significantly higher cell numbers after day 5, while the 10HA/PCL scaffold showed a slight advantage in terms of cell proliferation. Conversely, the 20HA/PCL scaffold had the lowest cell numbers among all groups and reached a plateau in cell proliferation by day 7. The results were in line with the observations from the fluorescent staining images and SEM images (Figure 8).

The effects of HA on cell proliferation studies have been extensively reported; the addition of HA generally exerts a positive effect on the enhancement of cellular activity of the scaffolds. This is because the addition of HA improves the hydrophilicity and increases the surface roughness of the scaffolds, which facilitates robust adhesion of cells to the scaffolds [42,43,75,76]. In this study, the incorporation of a moderate concentration of HA (5 wt% and 10 wt%) promoted cell proliferation, whereas an excessive amount of HA (20 wt%) led to a decrease in cell number. However, as seen from the SEM images in Figure 8b, the flattened cell shape and extended filopodia on the 20HA/PCL scaffolds were comparable to other scaffolds, suggesting that the reduction in cell number was not attributed to the alteration in the biocompatibility of the scaffold material. During the optimization of the HA/PCL powder process parameters, the sinterability of 20HA/PCL was consistently much lower than that of the other materials. The specimens of 20HA/PCL used for biocompatibility assessments were obtained with the optimized parameters. The SEM images of the 20HA/PCL composite powder (Figure 2b_4_) and the resulting cross-section of the scaffolds (Figure 7d) demonstrated that the addition of 20 wt% HA led to a loosened arrangement of HA particles on the surface of the microspheres, hindering their sintering and coalescence, thereby compromising both the actual HA content and the scaffold integrity. This accounts for the decrease in cell viability observed in the 20HA/PCL scaffolds. Therefore, it is crucial to control the HA content in the HA/PCL composite powders, as it not only affects the printability of the material but also the biological properties of the resulting scaffolds.

## 4. Conclusions

In this study, the HA/PCL composite powders were prepared for the SLS process using a modified S/O/W emulsion solvent evaporation method. The particle morphology, size distribution, powder flowability, and thermal and rheological behavior were evaluated. The HR values and flowability indices showed that the incorporation of HA nanoparticles improved the powder flowability as compared to pure PCL powder, while an excessive HA content (20 wt%) resulted in compromised flowability. The sintering window values of the prepared powders were wider than those of virgin PCL material due to the enhanced rearrangement capability of molecular chains during crystallization in solution. The addition of HA also led to an increase in melt viscosity. The addition of HA increased the compression modulus and yield strength of the specimens, with maximum achievable values improved from 129.8 MPa to 166.2 MPa and 20.2 MPa to 25.1 MPa, respectively. The porosity of the specimens varied between 3.64% and 22.84% depending on the processing parameters used. Cell adhesion and proliferation experiments demonstrated that the incorporation of HA improved the biocompatibility of PCL, which facilitated close cell adhesion to the scaffolds. It is worth mentioning that an excessive HA content led to insufficient sintering and coalescence of the PCL microspheres, thereby compromising the mechanical strength and integrity of the scaffolds and impacting their biological properties.

## Figures and Tables

**Table 1:** Process parameters for PCL and HA/PCL composite powders.

Process Parameters	PCL	5HA/PCL	10HA/PCL	20HA/PCL
Laser power *p* (W)	2.8–5.6	2.8–5.6	2.8–5.6	2.8–7
Laser scan speed *s* (mm/s)	3600	2400	2400	3600
Powder bed temperature (°C)	50	56	58	54
Layer thickness *t* (mm)	0.1	0.1	0.1	0.1
Hatch distance *h* (mm)	0.1	0.1	0.1	0.1

**Table 2:** The key flowability properties of the PCL and HA/PCL composite powders.

Sample	Angle of Repose (°)	Angle of Fall (°)	Bulk Density (g/cm^3^)	Tap Density (g/cm^3^)	Hausner Ratio (HR)	Flowability Index
PCL	35.2	18.3	0.57	0.66	1.158	79.5
5HA/PCL	25.4	14.2	0.67	0.70	1.045	97.0
10HA/PCL	25.7	19.2	0.64	0.73	1.058	96.0
20HA/PCL	29.9	18.5	0.69	0.77	1.203	81.5
PA12 [55]	/	/	0.48	0.54	1.125	/

**Table 3:** The key melting and crystallization properties of the virgin PCL, PCL, and HA/PCL composite powders.

Sample	*T*_im_ (°C)	*T*_m_ (°C)	*T*_ic_ (°C)	*T*_c_ (°C)	Sintering Window (°C)	Δ*H*_melt_ (J/g)	*x*_c_ (%)
Virgin PCL	53.9	64.5	38.4	34.0	15.5	83.1	59.6
PCL	58.9	63.8	39.8	37.5	19.1	82.1	58.8
5HA/PCL	58.8	63.2	40.1	37.8	18.7	81.1	58.1
10HA/PCL	58.6	63.2	40.5	37.8	18.1	76.9	55.1
20HA/PCL	58.7	63.5	40.5	37.8	18.2	76.1	54.5

**Table 4:** The key thermal stability properties of the PCL and HA/PCL composite powders.

Sample	*T*_d,onset_ (°C)	*T*_d,max_ (°C)	Δ*T* (°C)	*r*_d,max_ (%/min)	Residual Mass at 500 °C (%)
PCL	350.2	412.0	61.8	24.49	1.01
5HA/PCL	344.0	410.0	66.0	22.89	4.74
10HA/PCL	330.1	409.7	79.6	21.46	8.03
20HA/PCL	305.6	407.9	102.3	17.87	15.71

## Data Availability

The original contributions presented in the study are included in the article and Supplementary Materials, further inquiries can be directed to the corresponding authors.

## Supplementary Materials

The following supporting information can be downloaded at: https://www.mdpi.com/article/10.3390/polym16060731/s1, Figure S1: Effect of (a) PCL concentration, (b) emulsification speed on the particle size distribution of PCL; Table S1: The key properties for the flowability index of the PCL and HA/PCL composite powders; Table S2: The fitting parameters of the Carreau-Yasuda model for the virgin PCL, PCL, and HA/PCL composite powders.
