# Supplementary material for: Development of Hydroxyapatite/Polycaprolactone Composite Biomaterials for Laser Powder Bed Fusion: Evaluation of Powder Characteristics, Mechanical Properties and Biocompatibility
Source: Polymers (Basel). 2024 Mar 7;16(6):731. doi: 10.3390/polym16060731 (PMC10975002; doi:10.3390/polym16060731)
Supplement: Supplementary file 1 [file polymers-16-00731-s001.zip › polymers-2823607-supplementary.pdf]

polymers MDPI

Table S1. The key properties for the flowability index of the PCL and HA/PCL composite powders.

	Angle of Repose (°)	Angle of Spatula (°)	Compressibility (%)	Uniformity Coefficient	Flowability Index
**PCL**	35.21	52.5	14.0	2.8	79.5
**5HA/PCL**	25.38	28.5	4.0	2.7	97.0
**10HA/PCL**	26.69	26.6	5.0	2.4	96.0
**20HA/PCL**	29.98	41.3	17.0	4.0	81.5

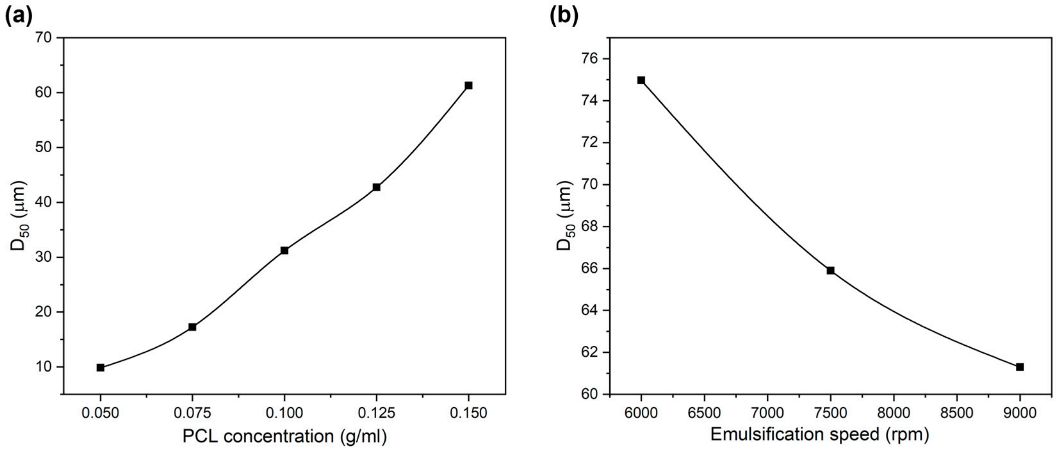

Figure S1. Effect of (**a**) PCL concentration, (**b**) emulsification speed on the particle size distribution of PCL.

Table S2. The fitting parameters of the Carreau-Yasuda model for the virgin PCL, PCL and HA/PCL composite powders.

	η_0	η_∞	λ	a	n	R^2
**Virgin PCL**	3304.3	63.8	70.6	55.9	0.52	0.995
**PCL**	5522.7	109.2	55.9	53.9	0.43	0.993
**5HA/PCL**	7817.6	197.7	30.1	37.8	0.35	0.992
**10HA/PCL**	9092.6	199.8	22.9	36.1	0.12	0.989
**20HA/PCL**	8177.0	122.1	23.3	28.2	0.23	0.996

The R^2-value is the goodness of fit.
